# An Inquiry into the Nature of Sleep and Death, with a View to Ascertain the More Immediate Causes of Death, and the Better Regulation of the Means of Obviating Them

**Published:** 1834-10-01

**Authors:** 


*An Inquiry into the Nature of Sleep and Death, with a View to ascertain the more immediate Causes of Death, and the better Regulation of the Means of obviating them.* Republished from the *Philosophical Transactions;* being the concluding Part of the Author's *Experimental Inquiry into the Laws of the Vital Functions.* By A. P. W. Philip, m.d., f.r.s. l. & e.—London, 1834. 8vo. pp. 254.

The work before us affords several useful lessons to the student of physiology, who will find in it striking examples both of what he ought to imitate, and what he ought to avoid. The papers it contains form the concluding part of Dr. Philip's researches into the laws of the vital functions; researches which have enriched science with many valuable facts, and conferred on their author a deservedly high rank among the physiologists of the day.

The perseverance which Dr. Philip has evinced in these important inquiries, his ingenuity in devising experiments, and his sagacity and caution in conducting them so as to ensure accurate results, are all deserving of the highest praise; but it must nevertheless be acknowledged, that his conclusions are often hasty, his reasoning inconsecutive, and, what is worse than all, that he frequently deserts the true path of experiment and observation, and loses himself in speculating on the merest assumptions. Several parts of the present work possess in full measure the characteristic excellencies of the author; but we are sorry to add, that others are in the highest degree obnoxious to the censure from which none of his productions can be entirely exempted: there are indeed some passages so idly theoretical, so utterly devoid of the genuine spirit of scientific research, that, if the name of Wilson Philip had not been attached to them, we should have been inclined to pass them by as altogether unworthy of attention.

We proceed to notice the papers in the order of their succession.

1. *On the Functions of the Nervous System, and the Relation which they bear to the other Vital Functions.* [From the *Philosophical Transactions* for 1829.]

This paper is intended as introductory to the rest, and consists of inferences from the facts which the author has at different times laid before the Royal Society.

Since we shall have occasion to differ materially from many of Dr. Philip's views,—nay, further, since some of his assertions are so unfounded, that it is difficult to believe that any physiologist could have soberly advanced them,—the reader will, we trust, excuse the copiousness of our extracts, and ascribe it to our desire to preclude misrepresentation, by giving the author's sentiments in his own words.

"The nerves," says Dr. Philip, "may be divided into two classes, those which proceed directly from the brain and spinal marrow to the parts to which they convey the influence of these organs; and those which enter such ganglions as receive nerves proceeding from different parts of the brain and spinal marrow, whether these nerves have or have not protuberances belonging to themselves, which have also been termed ganglions, but which receive only the different fibres that belong to the particular nerve to which they are attached, and from the circumstances in which they are placed, must have a different, or at least a more confined relation to other parts of the nervous system. To the former, therefore, I shall, for the sake of distinction, and to avoid circumlocution, confine the term ganglion.

"I beg leave to lay before the Society the following extract from lectures delivered by Mr. Brodie before the College of Surgeons, and which have not yet been published, in which this accurate anatomist and physiologist has given the sum of our knowledge respecting the structure of the ganglions.

"'Those bodies which are found in certain nerves which appear to be formed by an enlargement of the nervous substance, and which are denominated ganglia, are of a complicated structure. Into ganglia the nervous fibres may be traced, and from these ganglia the nervous fibres again emerge. Scarpa has paid much attention to the fabric of the ganglia, and he gives the following history of it. He says that the fasciculi of nervous filaments which enter a ganglion are separated and divided from each other, and that they are combined anew. A nervous fasciculus entering a ganglion divides into smaller fasciculi. These divide again, and cross and intersect each other at various angles. Then the divided fasciculi become again united, and as at first they divided into smaller and smaller fibres, so, when they begin to unite, they form gradually larger and larger bundles. At last the nerve which entered a ganglion emerges from it with its fibres collected into one or more fasciculi. Sometimes several nerves enter a ganglion, in which case they are all blended together, forming a complicated net-work, in which it is impossible to determine what belongs to one nerve and what belongs to another nerve. Every fasciculus or filament which enters a ganglion passes through it. There is no appearance of any one terminating in it.'

"'If we unravel the texture of a ganglion, we find that each nervous fibre retains its own peculiar neurilema; but, besides this, the spaces left between the intersection of the fibres are filled up with a peculiar soft substance of a greyish or yellowish colour. With the nature of this substance we are unacquainted. Some have considered it as corresponding to the cineritious substance of the brain and spinal marrow; but Scarpa is disposed to regard it as a soft cellular substance, filled with a greyish and mucilaginous matter in emaciated subjects, and with a yellowish oily matter in those that are fat.'" (P. 3.)

So far all is good; but, if the following passage does not startle the reader, we congratulate him on his equanimity:

"It is evident, from what has been said, that the ganglions and nervous plexuses which accompany them, resemble each other in their nature; and, as the nerves which terminate in then come from all, even the most distant parts of the nervous system, some from the brain, and some from the lower extremity, and all intermediate parts of the spinal marrow, we cannot help supposing that there is some design in thus uniting nerves from so many sources. One of the most striking differences between the ganglionic nerves and those proceeding directly from the brain and spinal marrow, is that, even independently of the ganglions and plexuses, the former every where freely anastomose, if I may borrow a term from the sanguiferous system; while the latter proceed in a more direct course, being less connected with each other in their progress, to the parts on which they bestow sensation and voluntary power, still further demonstrating the care with which Nature blends the ganglionic nerves. There is even reason to believe that the protuberances resembling ganglions belonging to individual nerves, serve the purpose of intimately combining the influence of the different fibres of the nerves they belong to, and that all the nerves having such protuberances contribute to the ganglionic system.

"What purpose is served by the perpetual intertwining of these nerves? It is impossible for a moment to conceive that it is without an object.

"This question is most likely to be answered by inquiring into the nature and functions of the parts supplied by them; those parts are the vital organs—namely, the thoracic and abdominal viscera, and the vessels even, as we shall find by experiment, where the parts are too minute to be made the subject of dissection, to their minutest ramifications.

"If the nerves proceeding from ganglions convey the influence of all the nerves which terminate in them, it would seem that, although to other parts the influence of only certain parts of the brain or spinal marrow is conveyed, the vital organs receive that of every part of them. This question can only be determined by experiment. That it must be answered in the affirmative appears from numerous experiments too simple to admit of our being deceived, which I made many years ago, and the details of which were laid before the Royal Society, and published in the Philosophical Transactions for 1815, and a few years afterwards, in my treatise on the Vital Functions. From them it appears that although the muscles of voluntary motion obey an agent affecting no part of the brain and spinal marrow but that from which their nerves take their origin, the heart is influenced by agents applied to every part of these organs, from the very uppermost surface of the brain and cerebellum to the lowest portion of the spinal marrow. The same was found to be the case with the blood-vessels to their minutest ramifications. Even the extremities of the arteries and veins, where they unite to complete the circulation, it was found, by the aid of the microscope, could be influenced, their action being either increased or impaired according to the nature of the means employed, nay, even finally deprived of power, by agents whose operation was confined either to the brain or spinal marrow; for on the power of the capillary vessels we shall find the circulation in a great degree depends.

"In some animals even of warm blood, as appears from experiments related in my treatise on the Vital Functions, the motion of the blood in the capillaries may be observed for two hours, or even more, after death, provided neither great and sudden injury to the nervous system, nor great loss of blood be occasioned, by the mode of death; that is, long after the heart has ceased to beat. The continued action of the capillaries appears from what is there said to be the cause of the large arteries being found empty some hours after death.

"It has also been shown, by experiments detailed in the same treatise, an account of some of which has appeared in the Philosophical Transactions, that the stomach and lungs are in like manner under the influence of both the brain and spinal marrow, their functions finally ceasing when they are deprived of any considerable portion of the influence they receive from either of these organs.

"The partial connexion with the nervous system of the organs supplied by the cerebral and spinal nerves, and the universal connexion with that system of those supplied by the ganglionic nerves, explain many of the phenomena, both of health and disease. Why are affections of the stomach and other vital organs instantly felt through every part of the frame, while the effects of those of a muscle of voluntary motion, or even an organ of sense, although often a part of greater sensibility, is confined to the injured part? If the eye or ear, or the muscle of a limb, be so deranged by a sudden blow, for example, as instantly to destroy its power, sight, hearing, or the voluntary power of the part is lost, and there the evil ends unless inflammation ensues; but a blow on the stomach, which instantly destroys its power, at the same moment destroys that of every other part. It is not difficult to answer the question, since the state of the stomach, from the cause just pointed out, may influence every part of the nervous system; and it appears from experiments, an account of which the Society did me the honour to publish in 1815, some of which were repeated by Mr. Cliff, that a powerful and sudden impression, made on any considerable part of this system, is capable of destroying the circulation by instantly depriving both the heart and blood-vessels of their power.

" Here the question naturally arises—For what purpose are the vital organs thus connected with every part of the brain and spinal marrow?

" This question is answered by experiments detailed in my treatise on the Vital Functions, an account of some of which appeared in the Philosophical Transactions for 1815 and 1822. From them it was found that the power of secreting surfaces is deranged by abstracting from them any considerable part of the influence either of the brain or spinal marrow; and, as the function of secretion is effected by the action of the nerves on the blood, as appears from experiments detailed in the papers just referred to, in another paper which I had the honour to lay before the Society, a few weeks ago, and from a greater variety of experiments detailed in my inquiry into the laws of the Vital Functions, it is evident that the presence of nervous power in a secreting organ would be useless, were not the blood on which it operates also supplied, and disordered if it were not supplied in due proportion; and, consequently, its supply varied as the supply of nervous power varies.

" We thus see not only why secreting surfaces are placed under the influence of every part of the nervous system, but also why it is necessary that the sanguiferous system should be under the control of the same laws which regulate the supply of the nervous power.

" It appears then that, by means of the system of ganglionic nerves, the influence of every part of the brain and spinal marrow is bestowed on secreting surfaces, and on those organs by which the supply of their fluids is regulated, and that the influence of every part of the brain and spinal marrow is necessary to their functions. But it is not the secreting power alone that is thus placed under the influence of every part of these organs; for it is a necessary inference, from experiments related in a paper which the Society did me the honour to publish, in their Transactions for 1827, (the next paper in this volume,) that the whole of those processes on which the healthy structure of the part depends are under the same influence.

" The influence, therefore, of the whole brain and spinal marrow is thus united by nerves from every part of these organs entering ganglions and plexuses, from which are sent to every part of the body nerves, proved by direct experiment to convey the influence of every part of them; and this combined influence of the brain and spinal marrow is employed in forming the various secreted fluids, and supporting the other processes on which the due structure of every part depends; and I have in more than one treatise pointed out how extensively the phenomena and cure of diseases are influenced by this cause." (P. 7.)

As soon as we had somewhat recovered from our astonishment at the affirmation that nerves from all parts of the brain and spinal marrow enter ganglions and plexuses, we had recourse to the list of errata, but without finding any relief; then we conned over the catalogues set forth by the chief bibliopolists of the day, to see if we could discover any work entitled "A New Anatomy of the Brain and Nerves, by Dr. Wilson Philip,"—no such thing; and since, on a further perusal of the volume before us, we find this novel announcement several times repeated in good set terms, we are reluctantly compelled to conclude that the author actually means what he says: we have therefore only to answer, in the words of Lactantius, (if it be admissible to quote one of the fathers of the church on such an occasion,) "Turpe est hominem ingeniosum dicere id, quod si neges, probare non possit." (*De Vera Sapientia*, c. 29.)

With respect to the theory involved in the passage we have quoted,—taking it exactly as it stands,—all that can be said is, that it rests on a most outrageous assumption, and is therefore not worth talking about. Without however asserting that nerves from every part of the brain and spinal marrow enter ganglions and plexuses, it may be supposed, and has been supposed by good physiologists, that the use of the ganglionic system is to combine the influence of the great nervous centres for the maintenance of the functions of organic life: but there is, we conceive, the following strong objection to this view of the subject. We have no proof that the greater part of the brain and spinal marrow are the seat of any other powers than those which preside over sensation and voluntary motion. It is true, indeed, as asserted by Dr. Philip, that the organic functions may be influenced through the medium of the nervous centres; but this proves nothing but that an intimate sympathy exists among the different parts of the nervous system, by which a cause operating on one part may influence any other part, or the whole. Now there is no reason to believe that the organic functions are sustained by the sensatory and voluntary powers, because the exercise of these functions is generally unaccompanied with sensation, and independent of the will. In supposing, therefore, that the ganglionic system associates the powers of the brain and spinal marrow, for the production of the phenomena of organic life, we should be adopting an hypothesis which, even if admitted to be true, would leave the functions in question entirely unaccounted for. Our author, indeed, in various parts of this treatise, speaks of the *vital* parts of the brain and spinal marrow, as contradistinguished from those which minister to sensation and voluntary motion; but he nowhere condescends to inform us where these parts are to be found.

There are, we think, better theories of the action of the ganglionic system than that under consideration; but, in the present imperfect state of our knowledge, the best way is to confess the truth—that we do not at all understand its functions, and to wait till the accumulation of more facts shall have placed us in better condition to reason on the matter.

Another important point treated of in this paper, is the relation of the sensorial power to respiration. The muscles by which the act of breathing is accomplished have generally been considered by physiologists as of the semivoluntary kind,—that is, as muscles which, though under the control of the will, are nevertheless capable of acting independently of it; and it has been regarded as a wise provision of nature, that a function so necessary to life should be continued automatically when the attention of the individual is entirely abstracted from it, or the influence of the sensorium suspended during sleep. Dr. Philip, however, takes a different view of the subject, and maintains that respiration is at all times a voluntary act.

"It has been customary to speak of the muscles of respiration as at least in part muscles of involuntary motion. What is meant by a muscle of voluntary motion? It is a muscle whose action, under all ordinary circumstances, we can excite, interrupt, retard, and accelerate at pleasure; but it is not a muscle whose action we can at all times control. There is no such muscle, because the impression on the sensorium tending to call any particular set of muscles into action may be so powerful, that we are unable to control it. Who can prevent the action of the muscles of the arm when fire is suddenly applied to the fingers? Neither do we mean by the term muscle of voluntary motion, one which we cannot call into action during sleep. If our posture during sleep becomes uncomfortable, we call the muscles both of the trunk and limbs into action for the purpose of changing it. The uneasiness caused by the continuance of the same posture sufficiently rouses the sleeper to make him will a change of posture, without rendering him at all more sensible to other impressions of a slighter nature, and his sleep continues.

"What muscles, then, are more under command than those of respiration? We can, on all usual occasions, interrupt, renew, retard, or accelerate their action at pleasure; and, if we cannot interrupt it for as long a time as that of the muscles of a limb, this depends on no peculiarity in the action of these muscles, but on the nature of the office they perform; and, if we excite them in sleep for the removal of an uneasy sensation, and cannot control them under a sense of suffocation,—that is, in a state of greater suffering than can be voluntarily borne, all this is no more than applies to every other muscle of voluntary motion: but, from the nature of our constitution, we must breathe many times every minute, and we need not turn ourselves more than once in many hours; a difference depending on circumstances which have nothing to do with the nature of the muscles we employ in either of these acts.

"If we find the breathing going on in apoplexy after all voluntary motion of the limbs has ceased, it is because the sensation exists which excites the patient to inflate his lungs, while there is none which excites him to move his limbs. In the slighter states of apoplexy, if the limbs be much irritated, the muscles which move them will also be called into action; and, in the severer states, if the patient breathes, when no irritation of the limbs can excite him to move them, it is because the want of wholesome air in the lungs, after a certain interval, produces a more powerful impression than any other means we can employ. People have voluntarily held the hand in the fire, but no man ever voluntarily abstained from breathing till the lungs were injured. When at length no irritation, however violent, can impress the sensorium, the breathing ceases, and death ensues. The mode of death in apoplexy sufficiently illustrates what is here said. We find the intervals of breathing becoming longer before it ceases. As the insensibility increases, a greater want of fresh air is necessary to excite the patient to inspire, till at length, the total privation of fresh air no longer producing any sensation, can no longer excite this effort.

"The muscles of respiration, then, it would appear, are as perfectly muscles of voluntary motion as those of the limbs, and are never excited but by an act of the sensorium. When there is no feeling to induce us to breathe, the breathing ceases." (P. 26.)

Now we are by no means prepared to assert, that all which is here advanced by our author may not be perfectly true; but, unfortunately, it is quite impossible to determine whether it be so or not. The only sure evidence we can have of any mental operation is *consciousness:* nay, this constitutes so essential a part of every such operation, that no act of the mind can be conceived to exist without it. In most instances the act of which the mind is conscious is sufficiently vivid, and of sufficient duration, to leave a permanent trace in the memory, and in these instances we are certain that such act of the mind has really taken place; but it is also conceivable that acts of the mind may sometimes occur, which are so slight and transitory, that, although the mind may have been conscious of them at the moment of their occurrence, no trace of such consciousness remains in the memory: they cannot, consequently, be recorded by the only person whose evidence is available, the individual in whose mind they took place; and hence we cannot have any actual proof of their existence, however probably it may be inferred from a process of abstract reasoning. The objection, then, we conceive, to Dr. Philip's hypothesis, is not that there is anything in it repugnant to reason, or at variance with any known fact, but that it is based on a doubtful point in metaphysics, which we have no means of resolving; and that, consequently, the hypothesis cannot be either proved or disproved.

We should always be extremely careful to avoid founding any physiological views on metaphysical reasoning. Some of the ablest physiologists of the last century have expended much learning and ingenuity in vain, from want of caution in this particular: we have a remarkable example in the writings of Whytt, a man of most extensive information and singular acuteness, but who, by continually referring vital phenomena to the intervention of the mind, where it was impossible, for the reasons above stated, to determine whether it intervened or not, has so embarrassed his reasoning, that his works are more apt to mislead than to instruct the student.

Dr. Milligan, the intelligent translator of Magendie, gives the following excellent precept on this subject: "Let the metaphysician always avail himself of the experiments of physiology as far as he is able, but let not the physiologist imagine that he can ever derive a reciprocal assistance from metaphysics."

We have noticed more particularly this opinion of Dr. Philip's, because, though it may be harmless enough as an hypothesis, he makes, as we shall presently find, a highly objectionable use of it, in reasoning on the causes of death.

2. *On the Effects of dividing the Nerves of the Lungs, and subjecting them to the Influence of Voltaic Electricity.* [From the *Philosophical Transactions* for 1827.]

The original and interesting experiments of Dr. Philip on the influence of galvanism in restoring the function of the stomach after the division of the pneumogastric nerves, are already familiar to the profession: his further inquiries have demonstrated analogous facts with regard to the lungs, and also brought to light another fact, having very important relations both to physiology and pathology.

"The Royal Society," says Dr. Philip, "did me the honour, in 1822, to publish the results of some experiments, from which it appeared that the secreted fluids of animals are so deranged by dividing the nerves of the secreting organs, and separating the divided ends, that those formed for the purposes of the animal economy are no longer capable of their functions; and that, after these functions are by such means destroyed, they may be restored by transmitting voltaic electricity through the secreting organs, by the portions of the divided nerves attached to them, the due properties of their fluids being thus restored.

"In the statement of these results, the attention was chiefly directed to the function of the stomach. In the present communication I shall make a few additional observations respecting the lungs.

"However much the secreting surface of the stomach may be deranged by the means just mentioned, its appearance, owing, we have reason to believe, to the extreme minuteness of its structure, is the same, or nearly so, as when the nerves have been left undisturbed; and, with the exception of occasional efforts to vomit, no symptom shows itself after the division of the nerves indicating the derangement of function which has taken place. Both in the symptoms and appearances after death, the derangement, occasioned in the lungs by the division of their nerves and the separation of the divided ends, is much more evident; the function as well as the structure of the lungs being, from their nature, more readily made the subjects of observation.

"Soon after the operation the animal begins to breathe with difficulty, and this symptom gradually increases, and is at length evidently the cause of death. On inspecting the lungs after death, the air tubes and cells, as far as they can still be traced, are found to contain a viscid fluid; and in considerable portions of the lungs, generally more or less according to the time the animal has survived the operation, every trace of both tubes and cells appears to be obliterated, the lungs both in colour and consistence assuming much of the appearance of the liver, and these portions of lungs sink in water; and, although examined with the greatest care, and the aid of a powerful magnifying-glass, both by Mr. Cutler, who was so kind as to give me his assistance, and myself, we could not perceive in them the least remains of the structure peculiar to this viscus.

"I wished, however, to ascertain, by means less fallacious than the sight, whether the change of structure in the parts most affected be such as to cause the total obliteration of their cavities, and Mr. Cutler, at my request, was so obliging as to make the following experiments, the account of which I shall give in his own words.

" 'If you cut out a portion of each of the eighth pair of nerves in the neck of a rabbit, it seldom dies within eight hours, and rarely survives more than twenty-four hours.

" 'On examination after death, the lungs are found, in many parts, covered with dark red patches.

" 'To ascertain the mischief done to the substance of the lungs, I endeavoured to fill them with mercury by the trachea, but, from the delicate structure of the air cells, a rupture took place, and the mercury escaped.

" 'I then endeavoured to inject the air cells through the trachea with the finest vermilion injection. In the healthy lungs the attempt was invariably successful, making the whole of a bright scarlet colour, and, on cutting into them, every part was found to be uniformly filled with the injection.

" 'After injecting the diseased lungs, the dark red patches remained on their surface: other parts of the lungs were of a bright red colour: some parts were partially injected, and other parts retained their natural appearance.

" 'This was explained on dissection. Those parts of the lungs which were completely injected had not suffered from disease, other parts had suffered sufficiently partially to obstruct the injection, while some parts were so completely hepatized that not a particle of injection could enter them, or the parts beyond them, which were not equally diseased.

" 'Those portions of the lungs which were completely injected sunk in water, from the weight of the injection.

" 'The hepatised portions, from their diseased state, sunk also, whilst the portions beyond them, having their natural appearance, floated.'

" If, as I have repeatedly ascertained, and various members of our profession have witnessed, after the nerves are divided, and the divided ends separated, the due degree of voltaic electricity be transmitted through the lungs, by those portions of the nerves which remain attached to them, no affection of the breathing supervenes; and the lungs, after death, are found quite healthy, unless the electricity has been applied of such power, or continues for such a length of time, as to excite inflammation, and then the appearances on dissection are those of inflammation, not those produced by the division of the nerves of the lungs.

" It appears, from these facts, that the effect of dividing the nerves of a vital organ, and separating the divided ends, is not merely that of deranging its secreting power, but all those powers on which its healthy structure depends; and that the effect of voltaic electricity is that of restoring all these powers. It is particularly to be observed, that the voltaic apparatus should be so arranged that its influence may be transmitted through the lungs as soon as the nerves are divided, the delay of even a short time appearing to give rise to more or less morbid appearance in the lungs." (P. 43.)

The disordered vascular action, and consequent change of structure in the lungs, from the interception of their communication with the encephalon, throws light on those cases in which disease of the brain induces secondary affections of the respiratory organs. It is by no means uncommon, for example, in cases of acute hydrocephalus, to find, on dissection, extensive disorganization of the lungs, where nothing in the early part of the case indicated the presence of disease in these organs. The analogy extends also to other organs at a distance from the brain, which may in like manner undergo rapid changes of structure, in consequence of disease of certain parts of the brain, which prevents their receiving a due share of nervous energy.

Such considerations as these ought to make us extremely cautious in our conclusions as to the degree of importance to be attached to those structural changes in the viscera frequently observed in fever, and other affections in which the centres of the nervous system have their functions manifestly disturbed. We should be careful lest we set down, as primary and essential, circumstances which may in truth be only consecutive, and which may have had no existence till towards the close of the disease, and no necessary connexion with it. But we are digressing. This paper, though short, is a model of physiological investigation: it contains nothing but facts accurately recorded, and conclusions legitimately derived.

3. *Some Observations relating to the Function of Digestion.* [From the *Philosophical Transactions* for 1829.]

The subject of digestion is one on which physiology is largely indebted to our author. It may be affirmed with truth, that nearly all that is known of the *mechanism* of this process is derived from his researches. His discovery of the possibility of substituting the galvanic power for the ordinary nervous influence in the production of the gastric fluid, is also one of the most original and important in modern physiology. The conclusion deduced by Dr. Philip from his experiments on this subject, namely, that galvanism is the agent actually employed in the living system for the accomplishment of the functions of the stomach, appears to us to be altogether invalid: this, however, does not at all derogate from the interesting nature of the fact, or the merit of the author in first bringing it to light.

Some experiments have been adduced by MM. Breschet and Milne Edwards, to prove that the same results may be produced by mechanical means, and that, consequently, the galvanic influence may act merely as a stimulant, by exciting a power of action still remaining in the nerves themselves, after their communication with the encephalon is cut off. Dr. Philip makes the following plausible objections to the inferences of these gentlemen.

"But it is maintained by some physiologists that the same effect may be produced by a mechanical agent.* They have related several experiments which appeared to them to prove, that when after a part of the eighth pair of nerves is removed, and thus the due secretion of gastric juice prevented, it may be restored by mechanically irritating the cut ends of the lower portions of the divided nerves. If such be the fact, it must materially influence our views, both with respect to the function of digestion, and the other secreting processes of the animal body.

"In judging of the result of such experiments, several things must be taken into the account, which appear to have escaped the attention of those gentlemen.

"At the time the animal is fed, in preparation for the experiment, there may be some food in the stomach, from previous meals, more or less digested, and there is always some gastric juice ready to act on any new food which may be presented to it. It is evident therefore, that, although the secretion of gastric juice cease at the moment of the excision of part of the eighth pair of nerves, some digested food must be found in the stomach for some hours after the operation; for, as I have ascertained, by numerous trials, many hours are required in such experiments for the stomach to propel into the intestine the remains of food previously digested, or that digested by the gastric juice previously formed.

"When therefore the contents of the stomach are examined in five or six hours, and generally even in ten or twelve, after the operation, more or less digested food is found lying next the surface of the stomach. But, when the animal survives the operation eighteen, twenty, or more hours, undigested food alone is found in it. The cause of so long a time being required wholly to expel the food which has undergone any degree of the digestive process, appears to be, that as digested food alone excites that action of the stomach which propels it into the intestine, and the more perfectly it is digested it excites this action the more readily, those parts of the digested food which have but imperfectly undergone the digestive process are expelled very slowly, so that it is very long before food wholly undigested alone is left.

* "See a paper entitled Mémoire sur le Mode d'Action des Nerfs Pneumogastriques dans la Production des Phenomènes de la Digestion. Par MM. Breschet et Milne Edwards (lu à la Société Philomatique, le 19 Fevrier, 1825.) —Extrait des *Archives Générales de Médecine*.

"That the longer the animal lives after the excision of part of the eighth pair of nerves, the less digested food is left in the stomach, is a fact now admitted by all who assisted at the experiments. Among the great number who have witnessed and been satisfied with their result, are Sir Humphry Davy, Mr. Thomas Andrew Knight, and Mr. Brodie, gentlemen whose experimental accuracy, in the opinion of the public, has never been surpassed.

"Of this fact the gentlemen to whose paper I have referred are not aware. They maintain, indeed, that the only effect on the digestive process produced by the excision of part of the eighth pair of nerves, is, that it becomes more tedious, being as perfect as when the nerves are entire, if a sufficient length of time be afforded. In speaking of the animals in which part of the eighth pair of nerves had been cut out, and comparing them with the healthy animal, they say: 'Enfin, si on laisse écouler un espace de temps plus grand encore entre l'operation et la mort des animaux, on pourra trouver que la digestion est complétement achevée dans l'un comme dans l'autre cas.'

"It will easily be perceived to what errors, respecting the effect on digestion, of depriving the stomach of the office of the eighth pair of nerves, this misconception, which probably arose from the animals employed in their experiments not having survived long enough to allow of the whole of the digested food being expelled from the stomach, must lead. Its effect was increased in the experiments referred to, by the different animals in each experiment having been confined to the same quantity of food. The most hungry would of course digest it fastest and most perfectly, because in them there would be the greatest quantity of gastric juice collected in the stomach before the excision of part of the nerves. To judge fairly of the result of the experiment the different animals must be allowed equally to satisfy their appetite, to eat till, from their manner of eating, it is found that the appetite has equally abated in all. It will be found, I think, that several other experimentalists have allowed themselves to be deceived by the circumstances here enumerated. We thus readily account for the discrepancies of writers. MM. Leuret and Lassaigne state that, in horses, eight hours after five or six inches of the eighth pair of nerves were cut out, the digestion was going on as usual,* while Mr. Field, whose experimental accuracy is generally acknowledged, and in whose experiments the same animals (horses) survived many times eight hours, found nothing in the stomach but undigested food covered with what appeared to be a layer of mucus, which I have often seen under the same circumstances in other animals.

"Such are the circumstances which I conceive misled those gentlemen who maintain that they can produce a sensible effect on the contents of the stomach by mechanical irritation of its nerves." (P. 53.)

* "Outlines of Human Physiology, by Herbert Mayo, F.R.S., second edition, pages 184 and 185."

Dr. Philip has repeated similar experiments, but with a different result.

"The mechanical irritation employed by MM. Breschet and Milne Edwards, in endeavouring to excite the digestive process after a portion of the eighth pair of nerves had been removed, was that of a thread attached to the cut extremities of the lower portions of the eighth pair of nerves and fastened to the neighbouring muscles, by which the motions of respiration kept the part in a state of constant irritation.

"In the third edition of my Inquiry into the Laws of the Vital Functions a similar experiment is related, in which the cut extremity of the lower portions of the nerves was fastened to a thread tied round the neck of the animal, by which it was in like manner kept in a state of constant mechanical irritation; yet in the stomachs of the animals after they had lived more than twenty hours, for the experiment was made more than once, nothing but undigested food was found. This experiment, with some others connected with it, was made publicly in the rooms of the Royal Institution, and all who felt an interest in the subject admitted to see the results, nor was there one who expressed a doubt respecting them.

"As, however, in these experiments the position of the nerves was more disturbed, and the thread was not applied, as in the experiments performed at Paris, Mr. Cutler, at my request, was so good as to make the following experiment. Three rabbits, after a fast of the same duration, were fed in the same way. In two of them a portion of each of the eighth pair of nerves was removed. The third rabbit was left undisturbed. In one of those in which the portions of nerve were removed, the cut end of the lower part of the nerves was, by means of a bit of thread, fastened to the neighbouring muscles, as in the experiments referred to. This rabbit died in ten hours, at which time the others were killed in the usual way.

"Mr. Cutler then took out the stomachs of all of them, slit them open, and laid them on the same plate; and Mr. Brodie was requested to examine and give his opinion respecting their contents, without having been told which was which. He at once pointed out the healthy stomach, the whole contents of which had undergone the action of the gastric juice. After carefully examining, and with an instrument moving about the contents of the other stomachs, he declared he could discover no difference in them. Both stomachs were chiefly filled with undigested food, the animals not having lived long enough after the operation for the expulsion of some imperfectly digested food that still remained in both.

"The foregoing experiments convinced those who witnessed their results, that the irritation caused by the attachment of the cut end of the nerves to the muscles, had no effect whatever in promoting the digestion of the food." (P. 59.)

"Since," observes our author, "as far as I know, no reply to the foregoing paper has, in the lapse of more than four years, been made, I infer that the gentlemen in question admit the accuracy of its conclusions."

Dr. Philip resumes at greater length the defence of his hypothesis, of the identity of the nervous influence with voltaic electricity, in the fifth paper contained in the present volume. We do not think that the discussion of this question would form a profitable occupation of the reader's time or our own: we shall therefore only remark, that the general opinion of physiologists is adverse to our author's conclusion, and that, where the facts of a case are equally accessible to all, the more prevalent opinion is usually the more correct one. We are the less inclined to argue the matter, because it has been already so amply and candidly considered by Dr. Bostock, that we cannot do better than refer the reader to the remarks of that excellent writer.*

4. *On the Sources and Nature of the Powers of Circulation.* [From the *Philosophical Transactions* for 1831.]

This paper contains some interesting and conclusive experiments on several points connected with the action of the blood-vessels. The first demonstrate the futility of the hypothesis which makes the resilience of the lungs an important agent in the circulation of the blood.

"It has been supposed," says Dr. Philip, "that what has been called the resilience of the lungs, that is, their tendency to collapse, by relieving the external surface of the heart from some part of the pressure of the atmosphere, is a principal means of causing it to be distended with blood, the whole weight of the atmosphere acting on its internal surface through the medium of the blood which is thus propelled from the veins into its cavities; and in this way it has by some been supposed that the motion of the blood through the whole of the venous part of the circulation is maintained. A similar effect has been ascribed to the act of inspiration, which, it is evident, must operate on the same principle; and this opinion has even been sanctioned by the Report of a Committee of the Royal Academy of Sciences of Paris, and in this country by men whose authority is deservedly high; and the effect of these causes, it is asserted, is increased by the elastic power of the heart itself." (P. 64.)

The following experiments are much to be preferred to any *à priori* reasoning on the subject, there being no science on which the logicians are more at fault than medicine, in all its branches: indeed, we scarcely know of a single instance in

* Elementary System of Physiology, vol. ii. chap. ix.

which the process facetiously called reasoning among doctors has led to anything but the conflation of big books, and the establishment of uncharitable relations between "dispassionate inquirers after truth."

"EXP. 1.—A rabbit was killed in the usual way for the table by a blow on the occiput, and the chest opened on both sides so as freely to admit the air. The lungs were then inflated eight or ten times in the minute by means of a pipe introduced into the trachea; the circulation was found to be vigorous. On laying bare one of the femoral arteries, it was observed to pulsate strongly; and on wounding it, the blood, of a florid colour, indicating that its colour had undergone the proper change in its circulation through the lungs, gushed out with great force; and, on introducing the hand into the thorax, the heart was found to be alternately distended and contracted, as in the healthy circulation.

"EXP. 2.—All the vessels attached to the heart in the newly dead rabbit being divided, and the heart removed, it was allowed to empty itself. Its contractions continued to recur, and in their intervals it assumed a perfectly flat shape, proving that the elasticity of the heart in this animal is so small, that it cannot even maintain the least cavity after the blood is discharged.

"It appears, from these experiments, that the circulation was vigorous when none of the causes to which the motion of the blood in the veins have been ascribed existed. In the first experiment, the chest being freely opened on both sides, so that the play of the lungs, on inflating them, could be seen, all effect on the heart, as far as related to the motion of the blood in the veins, either of the resilience of the lungs or the act of inspiration, was evidently prevented; and in the second, it was proved that no sensible elasticity of the heart existed; yet, while artificial respiration was performed, we could perceive no abatement in the vigour of the circulation." (P. 65.)

Having shown that the contractile power of the heart is one of the chief agents in the propulsion of the blood, our author successfully confutes the opinion of those who maintain that it is the sole agent; an opinion so irreconcilable with the most familiar observations, that it is only wonderful it should ever have been gravely supported. Did it ever happen to any of its abettors to see a young lady blush?—the interesting phenomenon is alone sufficient to decide the question against them.

"Not a few have ascribed, and even still do ascribe, the motion of the blood throughout the whole course of circulation to the contractile power of the heart alone, although it would not be difficult to prove, that to drive the blood through one set of capillary vessels, (for in man himself, in one important part of the circulation, it is carried through two, and in some animals through three, sets of capillaries before it returns to the heart,) I say it would not be difficult to prove that to drive it through one set of capillaries, at the rate at which the blood is known to move, would require a force capable of bursting any of the vessels. But here, as in the former instance, it is better to appeal to the evidence of direct facts than to any train of reasoning; and there is no want of such facts to determine the point before us, some of which I formerly had the honour to lay before the Society, and others are stated in my Treatise on the Vital Functions. The most decisive is, that the motion of the blood in the capillaries continues long after the heart has ceased to beat, and the animal in the common acceptation of the term is dead, and even in the warm-blooded animal, if it be favorably circumstanced, for several hours, and it is not for some time sensibly affected by the heart ceasing to beat; nor does this arise from some imperceptible impulse still given by the heart, because, when all the vessels attached to this organ are secured by a ligature, and the heart cut out, the result is the same." (P. 70.)

" If the circulation in the capillaries be thus independent of the heart, it is evident that the influence of that organ cannot extend to the veins. On comparing the whole of the foregoing circumstances, is it not a necessary inference, that the motion of the blood in the veins, like that in the capillaries, depends on the power of these vessels themselves? But, that we may not trust to any train of reasoning, where it is possible to have recourse to direct proof, I made the following experiment, with the assistance of Mr. Cutler.

" Exp. 3. In the newly dead rabbit, in which the circulation was maintained by artificial respiration, the jugular vein was laid bare for about an inch and a half; a ligature was then passed behind the part of the vessel nearest to the head, and the animal was so placed that the vein was brought into the perpendicular position, the head of the animal being undermost, so that it was necessary for the vein, in conveying its blood to the heart, to convey it perpendicularly against its gravity. The ligature, which was placed at what was now the lowest part of the exposed portion of the vein, was suddenly tightened, while Mr. Cutler and myself observed the vessel. The blood in the part of the vein between the ligature and the heart was instantly and completely expelled, as the transparency of the vessel enabled us to perceive. The vessel itself wholly collapsed, proving that all its blood had entered the heart, so that to a superficial view there seemed to be no vessel in the part where a large dark-coloured vein had just before appeared. In the mean time, on the other side of the ligature, the vein had become gorged with blood.

" In the foregoing experiment we see the blood rising rapidly against its gravity, where all causes external to the vessel on which the venous part of the circulation has been supposed to depend, had ceased to exist, and the vis à tergo was wholly destroyed by the ligature.

"By a similar experiment the power of the arteries in propelling the blood may also be demonstrated.

"Exp. 4. In a newly dead rabbit, the circulation being supported by artificial breathing, the carotid artery was laid bare for about an inch and a half. The animal was so placed as to keep the vessel in the perpendicular position, the head being now uppermost. A ligature was passed behind that part of the vessel which was next the heart, and Mr. Cutler and myself observed the vessel at the moment the ligature was tightened. The artery of course did not collapse, as the vein had done in the preceding experiment; but the blood was propelled along the vessel, so that it no longer appeared distended with it. It was at once evident, from the change of appearance in the vessel, that the greater part of the blood had passed on in a direction perpendicularly opposed to its gravity. It is worthy of remark, that the blood of the artery was propelled neither so rapidly nor so completely as that of the vein, the cause of which will be evident in the observations I am about to make on the nature of the function and powers of these vessels." (P. 72.)

Having proved very satisfactorily that both the arteries and veins possess an active power of propulsion, Dr. Philip concludes the paper by inquiring whether that power be of the muscular kind? and he concludes it to be so, because the blood-vessels appear to bear the same relation to the nervous system as the heart, which is on all hands acknowledged to be muscular. Having alluded to some of the ordinary arguments adduced by physiologists on each side of the question, our author proceeds as follows:

"I endeavoured, in papers which I had the honour to present to the Society, and which appeared in the Philosophical Transactions for 1815, to ascertain the relation which the heart bears to the nervous system, which is different from that of the muscles of voluntary motion. It appears from the facts there adduced, that this organ is not only, like the muscles of voluntary motion, independent of that system, although capable of being influenced through it either by means of stimulants or sedatives, and that even to the instantaneous destruction of its power; but that it equally obeys either set of agents, whether applied to the brain or spinal marrow, and to whatever part of these organs it is applied, provided it be to a part of considerable extent; while the muscles of voluntary motion obey no stimulus acting through the nervous system, unless it be applied to their nerves themselves, or to the particular parts of that system from which their nerves originate. I found, from repeated experiments, that the vessels bear the same relation to the nervous system as the heart does, their power being independent of this system, but equally with the heart capable of being influenced by either stimulants or sedatives applied to any considerable part either of the brain or spinal marrow, and that even to the instantaneous destruction of their power. They in all respects bear the same relation to the nervous system with the heart; the strongest presumption that their power is of the same nature.

"From the various facts stated or referred to in the foregoing paper, the following inferences appear to be unavoidable: that the circulation is maintained by the combined power of the heart and blood-vessels, and that the power of both is a muscular power." (P. 74.)

This is on the whole a very able paper, in all respects worthy of its author. If Dr. Wilson Philip would always write thus, he should be unto us a "magnus Apollo," and we should never fall out with any of his conclusions.

5. *On the Relation which subsists between the Nervous and Muscular Systems in the more perfect Animals, and the Nature of the Influence by which it is maintained.* [From the *Philosophical Transactions* for 1833.]

The first object of the author, in this paper, is to prove "that the brain and spinal marrow are the only active parts of the nervous system; the nerves, whether cerebral or ganglionic, with their ganglions and plexuses, being only the means of conveying, and, where the organs the functions of which require the influence of every part of the brain and spinal marrow, are concerned, combining the influence of the various parts of these organs." Our author states more at length his opinions as to the functions of the sympathetic nerve: on this subject, however, we have already stated our sentiments, which we need not here repeat. The following experiment, made by Mr. Field, at the request of Dr. Philip, is worthy of attention.

"Mr. Field partially divided the spinal marrow near the head in an ass in such a manner as to destroy the sensibility, as far as the experiment was concerned, but not to interrupt the respiration, thus bringing the animal into the best possible state for the experiment. It lay as still, and suffered as little, during it, as an animal quite dead in the usual sense of the word, while the circulation was more perfect than it could be under any artificial inflation of the lungs. In another respect, the state of the animal was particularly favorable, for he succeeded in exposing the semilunar ganglion and its plexuses with a very trifling loss of blood, not I believe four ounces. The heart was found to pulsate sixteen times in ten seconds, as ascertained by the pulsation of the arteries in the neighbourhood of the ganglion. The ganglion and its plexuses were then irritated by the point of the scalpel, and at length cut across in various directions; but, although the beats of the heart were repeatedly counted during these operations, they continued uniformly of the same fre quency. Spirit of wine was then applied to the wounded ganglion and plexuses, but without the least change in the beats of the heart. A strong infusion of tobacco in water was now applied, but with the same result, the beatings of the heart being still sixteen in ten seconds; nor could any variation in the force of the beats be observed in any part of the experiment.

"It appears, from this experiment, that we cannot influence the organs supplied by the ganglionic nerves by causes affecting the ganglions and plexuses, independently of the brain and spinal marrow; and the inferences from this and the preceding facts are unavoidable, that the former organs make only a part of the channel through which the influence of the latter is conveyed, and that the peculiar office of the ganglions and plexuses is to combine the influence of the nerves which terminate and are blended in them, and send off nerves endowed with their combined influence, in consequence of which the parts which receive the nerves proceeding from them become subject to every part of the brain and spinal marrow." (P. 92.)

It may be useful to compare this experiment with those of M. Magendie, showing the apparent inactivity of the great sympathetic in relation to sensation and voluntary motion.

"A ganglion," says M. Magendie, "may be pricked, cut, or torn, without the animal appearing conscious of it, and without any contractions taking place in the muscles. I have often tried these experiments on the cervical ganglia of dogs and horses; similar operations, if performed on the sensible nerves of the brain, would have produced horrible torture; if on the insensible, or those of motion, powerful muscular contractions. Let all the cervical and the first thoracic ganglia be removed, we do not perceive any sensible and immediate derangement in the functions even of those parts to which their nerves are distributed." (*Précis Elémentaire de Physiologie*, tome i. p. 214.)

It is curious enough to observe the very different conclusions at which these two physiologists arrive, from experiments whose rationale is similar, though their object be not the same.

From the apparent inactivity of the sympathetic with respect to certain functions dependent on the nervous system, Dr. Philip concludes that this nerve has no distinct function of its own, but conveys and combines the influence of the brain and spinal marrow. From a like inactivity with respect to certain other functions, also dependent on the nervous system, M. Magendie concludes that we know nothing about the functions of the sympathetic, and that we are even premature in designating it as a nerve at all. In matters so uncertain, the more cautious opinion is the one to be preferred.

In alluding to the sympathy of the nerves, Dr. Philip introduces an observation which would be highly important, if true.

"I take this opportunity of directing the reader's attention to a law of sympathy, which, as far as I know, has been overlooked, but which has an extensive influence in determining its effects, and, consequently, the phenomena and progress of disease. It appears, from what is said in the first paper in the present volume, that the functions of the animal body may be divided into two classes,—those of the sensitive, and those of the vital system. Now the individual organs do not sympathise equally with the organs of both these sets of functions. Some sympathise most powerfully with those of the sentient, and some with those of the vital functions. If we except the brain, whose extensive sympathies are necessary consequences of its functions, the stomach and liver are the organs of most extensive sympathies. Of these organs the former sympathises most with the sentient, the latter with the vital organs. Hence it is that affections of the stomach are more immediately felt in every part of the frame, while those of the liver have a greater influence on the course of disease. The liver being an organ of dull sensation, the share it has in influencing the course of many diseases has often been overlooked, few sensations being referred to the region of this organ, and thus the success of our plans of treatment greatly abridged. I beg to refer the reader to what is said of the sympathies of the liver, and the manner in which the course of various diseases is influenced by them, in my Treatise 'On the Influence of minute Doses of Mercury, combined with the appropriate treatment of various Diseases, and the Principles on which it depends." (P. 97.)

The reader will no doubt be surprised, on referring to our author's Treatise on Minute Doses of Mercury, to find a copious application of the hypothesis above advanced, but no further proof of it than the bare assertion! In answer to which, we beg to quote the words used by Dr. Philip himself, on another occasion: "It is to be recollected that here, as in other cases, the onus probandi rests with the asserter." We shall therefore leave the Doctor for the present to labour under the aforesaid *onus*, and content ourselves with simply denying that the liver sympathizes more with the vital organs than the stomach.

The remainder of this paper is occupied with a discussion on the opinion of Dr. C. W. Henry, respecting the relation of the nervous to the muscular fibre, and with Dr. Philip's favourite subject of the identity of the nervous and galvanic powers. The former question, under a somewhat different form, has already been argued *ad nauseam* by the physiologists of the last century, who might as well have saved themselves the trouble, because, till some anatomist shall have the dexterity to separate the two kinds of fibres in a muscle, without destroying or injuring either of them, all the physiologists on earth may dispute till the end of time, without being any the wiser.

With regard to the galvanic hypothesis, we have already given our reasons for not wishing to enter into the discussion of it.

### 6. *On the Nature of Sleep.*

[From the *Philosophical Transactions* for 1833.]

Dr. Philip remarks, that, " to understand the nature of sleep, we must determine the laws peculiar to each of those systems which have relation to that state, and the manner in which each is capable of influencing the other;" and again the Doctor says, " it is necessary therefore to a clear view of the state of the functions of the animal body in sleep, to determine the bonds of union between the sensitive and vital systems, at first view so distinct, which render their existence, except for a very limited time, inseparable." In these sentiments we entirely concur; but, as a knowledge of the above-mentioned laws, influences, and bonds of union, appears to us to involve nothing more nor less than an intimate acquaintance with the whole microcosm to its inmost recesses, we think that our author's statement of the preliminaries to the right understanding of sleep amounts simply to a declaration that the inquiry is, in the present state of our knowledge, a hopeless one.

Dr. Philip's general view of sleep may be illustrated by the following passage.

" It appears, from all that has been said, that in the sensitive system alone we find organs capable of exhaustion from all degrees of excitement, and the exhaustion of which is consistent with a state of health, namely, the nerves of this system and those parts of the brain and spinal marrow with which they are associated; but it is a necessary inference, from the facts stated in the last paper I had the honour to lay before the Society, that the former of these only obey the latter. To the latter alone therefore we must look for the exhaustion which is the immediate cause of sleep.

" The parts of the brain and spinal marrow which are associated with the nerves and muscles of the sensitive system, gradually, from the effect of the usual stimulants of life, suffer such a degree of exhaustion that those stimulants can no longer excite them; and their functions, unless stronger stimulants be applied, necessarily cease. Impressions from external objects, consequently, are no longer perceived, and therefore cannot produce their usual effects either on mind or body. Thus the expenditure of excitability in those parts of the brain and spinal marrow, and consequently in the nerves and muscles whose functions depend on them, being arrested, the vital functions still continuing, such an accumulation of it takes place in all these organs as again renders them sensible to the usual stimulants of life, and the activity of the sensitive system is restored.

"On the parts of the brain, and, in some animals, of the spinal marrow, as I have already had occasion to observe, which are associated with the nerves and muscles of the sensitive system, the mental functions depend. Hence the phenomena of dreaming, on which I shall make a few observations, immediately connected with the other parts of this paper, after considering the manner in which the vital, is influenced by the state of the sensitive, system in sleep.

"We are now to consider the effects of sleep on those organs which have no share in its production.

"One of the most important circumstances relating to the state of the sensitive system in sleep is that its exhaustion is never so complete as, under all circumstances, to prevent its excitement. On this alone it depends, we shall find, that sleep has no fatal tendency. The degree of sensibility which remains in sleep is the distinguishing mark between it and the torpor of disease. That sleep alone is healthy from which we are easily roused. If our fatigue has been such as to render it more profound, it partakes of disease, that is, as will appear more clearly from what I shall have occasion to say of the different species of apoplexy, the vital system partakes of the debility, or some cause is operating which prevents the restoration of the sensitive system.

"Distinct as the vital and sensitive systems are, we know that neither can long survive the other. In a paper which appeared in the Philosophical Transactions for 1829, I stated or referred to the facts which prove that in all modes of death, except the most sudden, arising from causes which so impress the nervous system as almost instantly to destroy all the functions, those of the sensitive cease several hours before those of the vital system. The animal only dies when his means of enjoyment and intercourse with the world which surrounds him, no longer exist. This consequence is constant, and never long delayed. It is necessary, therefore, to a clear view of the state of the functions of the animal body in sleep, to determine the bonds of union between the sensitive and vital systems, at first view so distinct, which render their existence, except for a very limited time, inseparable.

"That the sensitive cannot exist independently of the vital system, is evident, on the slightest consideration; but the dependence of the latter on the former is much less so. The facts stated in the paper just referred to prove, that in the more perfect animals, the function of respiration, being the only vital function which requires the co-operation of the sensitive system, is here the bond of union. It appears, from those facts, that the muscles of respiration are, in the strictest sense, muscles of voluntary motion, the excitement of which consequently depends on the powers of that system. When the power of sensation wholly ceases, we cease to breathe.

"So confused have been the ideas of physiologists on this part of the subject, that, to account for the continued action of the muscles of respiration, and their intimate connexion with the vital system, they have supposed a third class of muscles, partaking of the nature of both the others,—those of voluntary and involuntary motion, to which it has been alleged the muscles of respiration belong. If this be the case, these muscles must change their nature every instant, because they are the same muscles which are employed in a thousand other acts universally acknowledged to be mere acts of volition; and, on the other hand, when powerful causes impede the breathing, all the muscles of the trunk are employed in this function. Besides, the facts which have been laid before the Society prove not only that there is no such class of muscles as that here supposed, but that the laws of excitability are the same in all muscles; the difference between the muscles of voluntary and involuntary motion depending wholly on the nature of their functions, and the circumstances in which they are placed. The nervous influence, although equally capable of influencing both, is supplied to them in different ways, and for different purposes; the usual functions of the muscles of voluntary wholly, of involuntary motion in no degree, depending on that system. The action of the muscles of respiration continues during sleep, because the exhaustion of the sensitive system is not complete, and the causes which influence this system in their excitement continues in our sleeping as well as waking hours; and the same is true of all other muscles of voluntary motion, as far as the causes which induce us to excite them are applied. In the soundest sleep we move our limbs, if their posture be rendered uneasy. We are not obliged to guard against these causes in sleep, else the motions they would excite would quickly rouse us. Those of respiration are too gentle to produce this effect.

"The only change which takes place in the action of the muscles of respiration during sleep is, that, in proportion as the sensibility is impaired, they are necessarily excited less readily, and the act of respiration is thus rendered less frequent, a more powerful application of the cause being required; the consequence of which is, that when they are excited, the air is drawn in with greater force; hence, and from the relaxation which is apt to take place during sleep in the parts about the fauces, particularly in those advanced in life and those of relaxed habits, the cause of snoring. Thus, we generally observe that the snoring is the louder the slower the breathing, that is, the relaxation of the fauces being the same, the more profound the sleep. The loudest snoring I ever heard, so loud as to startle the attendants, was in the last ten minutes of the life of a person who died of a disease of the brain impairing the sensibility, and who only breathed three or four times during that space.

"The other changes observed in the vital system in sleep are evidently the consequence of the diminished frequency of respiration. This necessarily produces a proportional diminution in the frequency of the pulse; the properties of the blood being less frequently renovated in the lungs, it less readily excites the heart and vessels, and the diminished force of circulation is as necessarily attended with a diminished formation of the secreted fluids. The state of the vital organs, in its turn, influences the sensitive system, and thus the sleep is rendered more profound. While health continues, however, no change takes place in the vital powers to prevent the perfect restoration of those functions by which the animal is again fitted for intercourse with the external world, for, as appears from what has been said, these powers are never impaired in sleep, but only less readily excited." (P. 135.)

To this, as an explanation, we have only to object, that it explains nothing. That sleep is a state which occurs when the powers of animal life are partially impaired by exercise, and that, during such state, the vigour of these powers is restored by the continued activity of the organic functions, is a view of the subject so very obvious, that it is probable no physiologist could be found who entertained any other. But all this is a statement of what occurs, not an explanation of it. If, indeed, the brain were known to be capable of generating the nervous energy only up to a definite point, at which it ceased to act, from simple incapacity of further action, we should then have a tolerably good explanation of sleep, in saying that this state commences when the brain is so exhausted as to be no longer capable of maintaining the animal functions, and continues till the undisturbed actions of organic life have restored it to that degree of energy at which it becomes capable of resuming them. But, although sleep be evidently intended to repair the wasted energy of the nervous system, and, in a healthy frame, supervenes when necessary for this purpose, still it is clear, from observation, that there is no definite point at which the brain ceases to act from exhaustion, so as to induce sleep. This state frequently occurs when there is no considerable degree of cerebral exhaustion: thus, there are many individuals, in perfectly good health, who seldom assume the horizontal position for five minutes without falling asleep. Again, when it is necessary to rouse the attention to any subject, we can easily shake off the disposition to sleep, and remain in a state of activity for several hours, without any detriment to health. A man also may be awakened in the midst of a sound sleep, and suddenly resume the full activity of all his powers of body and mind. It is therefore, we apprehend, much more probable, on the whole, that the state of sleep causes the cessation of the brain's action, than that sleep itself occurs in consequence of such cessation. What may be the proximate cause of sleep, we, for our own part, have not the smallest idea; nor do we think it likely that physiologists will ever be able to attain any precise knowledge on this subject: it seems too deeply involved in the arcana of life, of which no more is known now than at the beginning of the world.

We ought, however, to pursue the subject as far as we can, and we should commence by a patient and careful examination of the *effects* of sleep on every function of the mind and body which can be made the subject of distinct observation: in short, there is only one true way of investigating any subject, which is, first to observe, and to try, and afterwards to draw conclusions when the data are sufficient—not till then.

### 6. *On the Nature of Death.*

[From the *Philosophical Transactions* for 1834.]

We shall not detain the reader long with Dr. Philip's theory of death; for, to say sooth, we do not think it worthy of any particular attention. Natural death—that is, death from old age, differs from sleep, according to our author, only in degree. In sleep, sufficient sensorial power is retained to keep up respiration: in death, the sensorial power is so much diminished, that the uneasy sensation which causes the voluntary act of respiration during life no longer produces its wonted effect, and accordingly the man dies,—not, as the old adage has it, for want of breath, but for the want of the will to breathe. This is a very short way of disposing of the subject, and the theory has the advantage of simplicity, whatever else it may lack. We have, however, before endeavoured to show that Dr. Philip's view of the voluntary character of respiration is, from its very nature, insusceptible of proof; and his theory of death being mainly based upon it, both must fall to the ground together.

On viewing our author's general position, that sleep and death differ only in degree, the casuists might be apt to object, that where two things stand in the relation of cause and effect, the degree of effect produced is, *cæteris paribus*, precisely commensurate with the intensity of the cause, and that, consequently, since the effect of sleep is known to consist in an increased activity of all the powers of life, the effect of death, which is merely a greater degree of sleep, ought to consist in a more than usually exuberant vitality. But the fact is, the author is obliged to recede from his position, and, after having asserted that sleep and death are one and the same in kind, and different only in degree, he proceeds to explain a circumstance in which they differ very widely in their nature.

In natural sleep, it will be remembered, according to Dr. Philip's view of the subject, the continued activity of the organic functions has the effect of rekindling those of the animal life: hence, to account for the final extinction of the latter in death, we must presuppose a failure in the former. This our author admits, thereby also in reality admitting a fundamental difference between the two states.

"The state which immediately precedes the last act of dying, then, according to the common acceptation of the term, and sleep, depend on a failure of function in the same organs. In what, then, consists the difference of these states? The most evident is, that the one is a temporary, the other a final failure; and it will appear, that in the only death which can strictly be called natural, the state of the sensitive system which immediately precedes death differs from its state in sleep in no respect but in degree.

"The cause of sleep, as appears from the paper above referred to, is uniformly the same, a diminished excitability of the sensitive parts of the brain and spinal marrow, in consequence of the action of the ordinary stimulants of life; but a loss of excitability in those parts, we shall find, is never the sole cause of death, and often makes no part of its cause. In sleep, we have seen that the sensitive parts of the brain and spinal marrow regain their functions in consequence of the continued vigour of the vital system, by which their excitability is restored. To render the exhaustion which constitutes sleep permanent, therefore, the powers of this system also must fail; and, if any cause of failure in these powers occur, it is evident, that whatever be the state of the sensitive system, its powers must fail with them.

"The natural death of the animal is the death of old age; and as this is the simplest form of death, it is that which I shall first consider. We shall find that the state which immediately precedes this death, and must consequently be considered as its cause, must, in the nature of things, differ from sleep in no other respect than the less vigorous state of the functions of both systems, and consequently that these states are identical; the greater or less general vigour making no difference in their nature.

"We are not necessarily born to suffering. All natural states, with the exception of child-bearing, (and in its most natural state even this is hardly an exception,) are more or less pleasurable. It will appear, from the nature of our constitutions, that the last feelings in natural death are necessarily of the same nature as those which precede sleep. It is only where the course of our decay is disturbed, that suffering of any kind attends it.

"From a knowledge of the animal economy, we might, independently of experience, have foretold that a state of sleep would be that which immediately precedes the last act of dying from old age.

"It appears, from what was said of the nature of sleep, in the paper above referred to, that although the vital organs do not, in it, partake of the peculiar state which constitutes sleep, their functions are all, for the time, impaired by the exhaustion of the sensitive system. The respiration, we have seen, is rendered less frequent, in consequence of which the activity both of the circulation and the other assimilating functions which depend on it, is, for the time, lessened.

"Now, as the death of old age arises from the gradual failure of those functions, it must necessarily take place at the time at which their vigour is most impaired. If the vital powers are still capable of restoring the sensitive system under the disadvantage of a diminished frequency of respiration, it is evident that, if their decay be gradual, nothing occurring suddenly to accelerate it, they cannot fail to maintain the functions of that system during the short time which intervenes before the recurrence of sleep again exposes them to the same difficulty. Their failure necessarily takes place at the time when their functions are most difficult. The death of old age, therefore, is literally the last sleep, uncharacterised by any peculiarity. The general languor of the functions in the last waking interval is attended with no peculiar suffering, and the last sleep commences with the usual grateful feelings of repose, the last feelings experienced; for, with what takes place after them, the feelings, being suspended, have no concern.

"The only difference between the last, and the sleep of former times, is, that the exhaustion of the sensitive system, which is at first, as in the latter case, only partial, (for in the beginning of that sleep the sleeper may be roused by more powerful stimulants than those which preceded it,) becomes in its continuance, in consequence of the failure of those powers which formerly restored the sensitive system, complete." (P. 163.)

But the impaired state of the organic powers, which Dr. Philip admits to be essential to death, remains unaccounted for. On recurring to the paper on Sleep, we find it distinctly maintained that the muscles engaged in the organic functions are not liable to have their power exhausted in *any degree* by natural and healthy stimuli.

"But the muscles employed in the vital functions obey a better regulated stimulant, which never, except in disease, produces any degree of excitement that impairs their power. In many diseases, we see the effect of such excitement. If it does not abate soon, and we cannot by artificial means in a short time reduce it, death is always the consequence: and even a short continuance of it produces a degree of debility that so impairs the powers of life as to render their restoration both slow and difficult. *Thus it is evident, that on the capability of the muscular fibre to be moderately excited,*

*without suffering any degree of exhaustion, life immediately depends.*" (P. 132.)

Whence then the exhaustion of the organic powers which incapacitates them for the renewal of the animal functions, when the term of natural life is concluded? The truth is, that the organic powers *are* susceptible of exhaustion from the ordinary stimuli of life, though more gradually and less perceptibly. All that we know about death from old age is, that, when the entire organism is worn out, the man dies; and Dr. Philip's theory of death amounts to little more than a circumlocutory, and somewhat contradictory, statement of this familiar fact.

We have already severely taxed the patience of the reader, and must hasten to conclude. In saying that Dr. Philip has, in many parts of this volume, indulged in speculations so idle, that they would be barely excusable in the thesis of a nascent doctor; in saying, moreover, that he is very frequently at variance with himself, we do not, in effect, derogate anything from his knowledge or ingenuity as a physiologist, though perhaps somewhat from his sound judgment as a philosopher; because, in asserting that he has most signally failed, in his attempts to elucidate several subjects, we merely assert that he cannot do what is impossible,—that he cannot reason to the purpose without sufficient data.

"Magnis tamen excidit ausis!"

The besetting sin of Dr. Philip is presumption, which, in some parts of this work, is carried to a height absolutely ludicrous. It is impossible to read without a smile the passage above quoted, in which he describes what *must* be the agreeable sensations of a man dying by the extinction of his sensorial powers. Persons in this comfortable condition are usually particularly incommunicative; there is very little oral tradition to assist us; and hence, the only qualification for describing such a state would consist in having passed through it,

"Visendus ater flumine languido
Cocytus errans:"

and, accordingly, if this paper, instead of being "Observations on the Nature of Death, by Dr. Wilson Philip," had been "Practical Illustrations of the Process of Dying, by a Disembodied Spirit," we should have listened with much awe and deference to the ghostly instructor: as it is, we conclude that Dr. Wilson Philip knows precisely as much about the matter—as we do ourselves.

In parting with Dr. Philip, we would, in sincere goodwill, remind him that the highest reputation may be trifled away. His own has been well earned, and long may it last; but, if he has nothing in store better than these treatises on Sleep and Death, we advise him by all means to slumber on his laurels.

